# Atypical Pruriginous Pustular Eruption Preceding Locally Advanced Rectal Cancer: A Case Report and Gut–Skin–Tumour Axis Hypothesis

**DOI:** 10.3390/diagnostics16111592

**Published:** 2026-05-22

**Authors:** Monica Manciulea (Profir), Luciana Alexandra Pavelescu, Sanda Maria Crețoiu

**Affiliations:** 1 Department of Morphological Sciences, Cell and Molecular Biology and Histology, Carol Davila University of Medicine and Pharmacy, 050474 Bucharest, Romanialuciana.pavelescu@umfcd.ro (L.A.P.); 2 NutriMedX—Functional and Regenerative Medicine, Dacia 51 Blvd., 010406 Bucharest, Romania

**Keywords:** rectal adenocarcinoma, paraneoplastic dermatosis, gut–skin axis, faecal immunochemical test, intestinal barrier dysfunction, long-course chemoradiotherapy

## Abstract

**Background and Clinical Significance:** Cutaneous paraneoplastic phenomena are infrequently characterised in colorectal cancer (CRC), and chronic pruriginous inflammatory eruptions in particular have received limited attention. In older adults, persistent treatment-resistant dermatoses of unclear aetiology may represent overlooked extraintestinal diagnostic clues to occult malignancy, including potentially curable CRC. Faecal immunochemical testing (FIT) for occult bleeding is a low-cost, non-invasive tool whose role outside conventional alarm-symptom triage remains underexplored. **Case presentation:** A 72-year-old woman presented for outpatient evaluation with several months of pruriginous, pustular, and crusted symmetric eruption involving the dorsal aspects of the limbs, refractory to standard dermatologic treatment, and without gastrointestinal symptoms. A non-invasive systemic stool-based work-up demonstrated detectable faecal haemoglobin (iFOBT), mildly elevated faecal calprotectin (51.6 mg/kg, ULN 50 mg/kg), markedly elevated faecal alpha-1-antitrypsin (631 µg/mL; 2.3× ULN), and predominance of Escherichia coli on stool culture. Colonoscopy revealed a locally advanced rectal adenocarcinoma; staging classified the lesion as cT3N1M0. The patient received long-course neoadjuvant chemoradiotherapy (50 Gy, concurrent capecitabine) followed by low anterior resection with total mesorectal excision and pathological complete response (ypT0N0, R0), and adjuvant capecitabine. The cutaneous eruption resolved progressively in parallel with antineoplastic therapy without specific dermatologic intervention. The patient remains in remission at over 36 months. **Conclusions:** Persistent, unexplained, treatment-resistant pruriginous/pustular cutaneous eruptions may, in selected patients, coincide with an underlying malignancy, including colorectal cancer, and should prompt careful individualised clinical assessment, including review of age-appropriate colorectal cancer screening status. This single case raises the hypothesis that quantitative faecal immunochemical testing (FIT) may be prospectively evaluated as a low-cost, non-invasive triage tool in carefully selected patients aged ≥50 years with persistent dermatoses of unclear aetiology, even in the absence of gastrointestinal symptoms. Positive FIT results should be managed according to established local colorectal referral pathways. NICE diagnostics guidance DG56 supports FIT use in symptomatic adults with suspected lower gastrointestinal pathology; however, any extension of FIT to extraintestinal presentations remains investigational and requires formal validation through prospective studies assessing diagnostic yield, cost-effectiveness, and stage distribution.

## 1. Introduction

Colorectal cancer (CRC) is the third most commonly diagnosed malignancy and the second leading cause of cancer death worldwide [1,2]. Although screening programmes have improved early diagnosis, a substantial proportion of patients still present at advanced stages, particularly in the absence of overt gastrointestinal alarm symptoms [3,4]. Diagnostic delay is a recognised determinant of unfavourable prognosis [5], and clinically inconspicuous heralding signs—including extraintestinal manifestations—therefore deserve heightened attention.

Cutaneous paraneoplastic syndromes encompass a heterogeneous group of dermatoses that may precede, parallel, or signal the recurrence of an underlying neoplasm [6,7,8,9]. Curth’s postulates remain the conceptual reference for establishing such an association, particularly the temporal coincidence between tumour and dermatosis and their parallel evolution under treatment [10]. Several classical paraneoplastic dermatoses have been described in the context of solid tumours, including CRC [11,12,13]; however, atypical, non-classical pruriginous and pustular eruptions in CRC remain poorly characterised. A recent narrative review has explicitly raised the possibility that the gut–skin axis may contribute to such manifestations [14], and growing evidence on the gut microbiome, intestinal barrier function, and systemic inflammation [15,16,17,18,19] supports the biological plausibility of this concept.

A second, less well-defined category includes non-specific inflammatory, eczematous, pruriginous, or pustular eruptions that lack pathognomonic clinical or histopathological features and may overlap with common benign dermatoses. This category is particularly prone to delayed recognition, especially when gastrointestinal symptoms are absent.

Faecal immunochemical testing (FIT) for occult bleeding is a low-cost, non-invasive tool supported by the 2023 NICE diagnostics guidance DG56 for use in symptomatic adults with suspected lower gastrointestinal pathology [20]. Its potential role outside established colorectal referral pathways, including in patients with unexplained extraintestinal manifestations, remains investigational and requires prospective evaluation. In this context, FIT should not be regarded as a validated diagnostic strategy for dermatologic presentations, but rather as a possible triage tool whose clinical utility in selected atypical scenarios remains to be defined.

We report the case of a 72-year-old woman in whom a persistent, treatment-resistant pruriginous/pustular cutaneous eruption preceded the diagnosis of locally advanced rectal adenocarcinoma. Faecal haemoglobin positivity, identified during outpatient evaluation prompted by the unexplained dermatosis rather than by a pre-existing routine screening programme, led to colonoscopic diagnosis. The patient subsequently achieved a complete pathological response and remains in remission at over 36 months. The cutaneous eruption improved in parallel with tumour-directed therapy and without specific dermatologic intervention; however, in the absence of skin histopathology and mechanistic validation, a causal paraneoplastic relationship cannot be established.

This case is presented as a hypothesis-generating clinical observation and as a cautious illustration of a possible gut–skin–tumour relationship, without claiming a demonstrated mechanism. We further discuss the diagnostic uncertainty, the limitations of the available dermatologic and microbiological data, and the rationale for prospectively evaluating FIT-based triage only in carefully selected older adults with persistent, unexplained, treatment-resistant dermatoses.

## 2. Case Presentation

### 2.1. Patient and Clinical History

**Figure 1:**
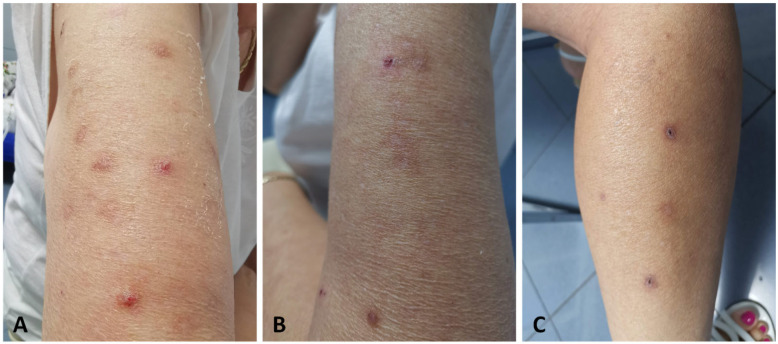
Clinical photographs of the cutaneous eruption at first outpatient evaluation. Symmetric pruritic, pustular, and crusted eruption involving the dorsal aspects of the forearms (**A**–**C**) and the lower legs. Note the predominantly extensor distribution and the lack of nodular thickening typical of prurigo nodularis. The patient has provided written informed consent for the publication of these images in anonymised form.

A 72-year-old Caucasian woman, hypertensive and previously thyroidectomised under stable substitution, was referred for outpatient internal medicine-oriented evaluation in October 2021 because of a chronic, intensely pruritic, pustular, and crusted cutaneous eruption involving symmetrically the dorsal aspects of the forearms, hands, lower legs, and dorsa of the feet. The patient described a 5-month evolution, with progressive aggravation despite multiple sequential dermatologic treatments including topical corticosteroids, oral antihistamines, topical antiseptics, and a course of oral antibiotics, none of which induced sustained improvement. Pruritus disturbed sleep. There were no constitutional symptoms, no weight loss, no abdominal pain, and no change in bowel habit. The patient reported no household contacts with similar lesions and no exposure to arthropods, novel cosmetics, or topical sensitisers. Personal and family history were non-contributory for atopy or autoimmune skin disease. Clinical photographs (Figure 1A–C) document the eruption. Because no skin biopsy was obtained, the cutaneous condition is described throughout the manuscript in morphological terms rather than as a definitive dermatologic entity.

At the time of the initial outpatient evaluation, no definitive dermatologic diagnostic procedure had been performed. Specifically, skin biopsy, histopathological examination, direct immunofluorescence, dermoscopy, and microbiological culture of the skin were not available. No eosinophil count, total IgE, or inflammatory marker dataset was sufficiently complete to support a specific immunologic diagnosis. Therefore, the clinical reasoning relied on morphology, distribution, lack of exposure history, failure of conventional dermatologic therapy, and subsequent parallel evolution with cancer-directed treatment.

### 2.2. Outpatient Stool-Based Work-Up

Given the persistence of the eruption despite repeated dermatologic management and the absence of an identifiable external trigger, a non-invasive systemic work-up was undertaken (Table 1). This was not part of a pre-existing routine colorectal cancer screening programme; it was initiated because the eruption remained unexplained after conventional dermatologic management. Stool analysis demonstrated detectable faecal haemoglobin (immunochemical faecal occult blood test, iFOBT), mildly elevated faecal calprotectin (51.6 mg/kg; reference ≤ 50 mg/kg), markedly elevated faecal alpha-1-antitrypsin (631 microg/mL; reference ≤ 270 microg/mL; ~2.3× upper limit of normal), preserved pancreatic elastase, and preserved secretory IgA. Microbiological analysis revealed an increased *Escherichia coli* load (1 × 10^7^ CFUs/g; reference ≤ 1 × 10^6^), increased alpha-haemolytic streptococci, and absence of yeasts and moulds. These microbiological findings were interpreted cautiously as non-specific stool culture abnormalities rather than as a comprehensive microbiome profile. Faecal haemoglobin positivity was the finding that prompted urgent referral for colonoscopy.

### 2.3. Endoscopic and Histopathological Diagnosis

Total colonoscopy (November 2021) revealed an ulcerated, partially circumferential, friable lesion in the upper rectum, approximately 12 cm from the anal verge. Biopsies established the diagnosis of moderately differentiated invasive rectal adenocarcinoma. Immunohistochemistry showed retained nuclear expression of MLH1, MSH2, MSH6, and PMS2, indicating mismatch repair (MMR)-proficient status. Ki-67 proliferation index was approximately 80%. Pelvic magnetic resonance imaging staged the lesion as cT3N1 with mesorectal nodal involvement; computed tomography of the thorax and abdomen, performed as part of oncological staging, excluded distant metastases. No dedicated pre-colonoscopy whole-body paraneoplastic screening algorithm was applied; investigations were guided by the positive faecal haemoglobin result and subsequent rectal cancer diagnosis.

### 2.4. Multidisciplinary Management and Treatment Course

Following multidisciplinary tumour board discussion (December 2021)—the patient declined enrolment in a contemporaneous total neoadjuvant therapy protocol and elected for standard long-course chemoradiotherapy followed by surgery, in keeping with the management options available at that time [21,22]—neoadjuvant chemoradiotherapy was administered between 14 December 2021 and 17 January 2022, consisting of pelvic intensity-modulated radiotherapy (50 Gy in 25 fractions) with concurrent oral capecitabine (825 mg/m^2^ twice daily on radiotherapy days). Treatment was well tolerated, with no grade ≥ 3 adverse events. Restaging MRI demonstrated significant tumour regression. Low anterior rectal resection with total mesorectal excision was performed on 9 March 2022.

### 2.5. Pathological Response and Adjuvant Therapy

Pathological examination of the surgical specimen demonstrated a complete pathological response (ypT0N0, R0; Ryan tumour regression grade 0), with no viable tumour cells identified either at the primary tumour site or in the examined lymph nodes (0/8). Although fewer than 12 lymph nodes were retrieved, reduced lymph node yield is commonly observed after neoadjuvant chemoradiotherapy for rectal cancer and has been documented in systematic reviews and meta-analyses [23]. Adjuvant capecitabine monotherapy (1250 mg/m^2^ twice daily, 14 days on/7 days off, for 8 cycles) was completed between April and October 2022. The patient tolerated adjuvant therapy without significant toxicity.

### 2.6. Cutaneous Evolution and Parallel Resolution

**Figure 2:**
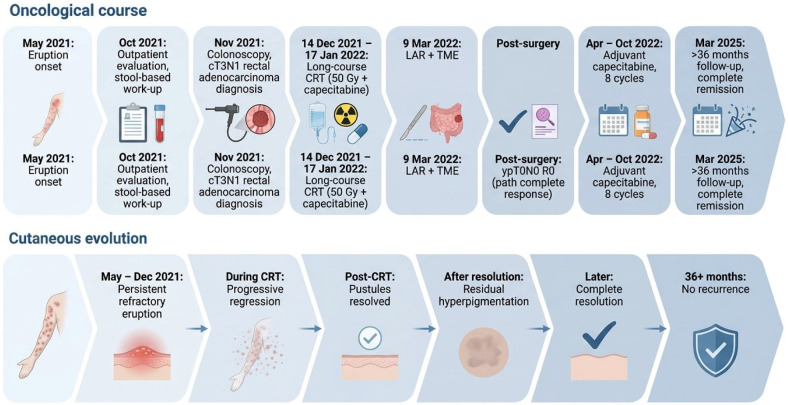
Parallel oncological course and cutaneous evolution. Schematic timeline illustrating the temporal relationship between the diagnosis and treatment of rectal adenocarcinoma and the progressive resolution of the cutaneous eruption. The upper row summarises the oncological course, from the onset of the eruption in May 2021, outpatient stool-based evaluation in October 2021, colonoscopic diagnosis of cT3N1 rectal adenocarcinoma in November 2021, long-course chemoradiotherapy with capecitabine between December 2021 and January 2022, low anterior resection with total mesorectal excision in March 2022, complete pathological response after surgery, adjuvant capecitabine from April to October 2022, and complete oncological remission at more than 36 months of follow-up. The lower row summarises the parallel cutaneous course, showing persistent refractory eruption before cancer diagnosis, progressive regression during chemoradiotherapy, resolution of the pustular component after treatment, residual post-inflammatory hyperpigmentation, complete clinical resolution, and absence of recurrence during follow-up.

In parallel with the oncological treatment, the cutaneous eruption underwent progressive, sustained regression. By the end of neoadjuvant chemoradiotherapy, pruritus had subsided and the pustular component had resolved. Residual post-inflammatory hyperpigmentation persisted for several months and faded gradually. No specific dermatologic treatment was administered during oncological therapy. The strict temporal coincidence between tumour-directed treatment and resolution of the dermatosis—without dermatologic intervention—is compatible with a possible paraneoplastic relationship according to the temporal and parallel-course components of Curth’s criteria [10], but causality cannot be established in the absence of skin histopathology and mechanistic validation. The parallel oncological and cutaneous courses are summarised in Figure 2.

### 2.7. Long-Term Follow-Up

The patient remains in complete oncological remission at over 36 months of follow-up (last surveillance visit: March 2025), with normal carcinoembryonic antigen, normal cross-sectional imaging, and normal endoscopic surveillance. The cutaneous eruption has not recurred, and the patient reports no pruritus.

## 3. Discussion

### 3.1. Cutaneous Paraneoplastic Phenomena and Curth’s Criteria

Cutaneous paraneoplastic syndromes are a heterogeneous group of dermatoses, summarised in Table 2 [6,7,8,9,11,12]. Their attribution to an underlying tumour is conventionally assessed against Curth’s criteria, which favour temporal coincidence between tumour and dermatosis, parallel evolution under tumour-directed therapy, statistical association, exclusion of common alternative aetiologies, and (when feasible) histopathological correspondence [10]. The present case satisfies only some of these criteria, particularly temporal association and parallel improvement: the eruption preceded the diagnosis, was refractory to dermatologic intervention, resolved progressively under antineoplastic therapy without specific dermatologic treatment, and has not recurred at over three years of follow-up. However, the lack of skin biopsy, direct immunofluorescence, dermoscopy, skin culture, and formal cutaneous immunologic profiling prevents definitive nosological classification. The classical paraneoplastic dermatoses described in CRC [13,24,25,26] do not match the pruriginous, pustular, symmetric phenotype documented here. Accordingly, we interpret this case as a possible, non-classical paraneoplastic eruption rather than as an established CRC-associated dermatologic entity.

Non-classical inflammatory eruptions are especially difficult to attribute to malignancy because they overlap clinically with common dermatoses and are often recognised, if at all, only retrospectively after tumour diagnosis and treatment response.

### 3.2. The Gut–Skin–Tumour Axis as an Interpretive Framework

The gut–skin axis describes the bidirectional relationship between intestinal microbiota, mucosal barrier function, and cutaneous inflammation [15,16,17]. In CRC, tumour-associated dysbiosis, including enrichment of *Fusobacterium nucleatum*, pks+ *Escherichia coli*, and enterotoxigenic *Bacteroides fragilis*, as well as impaired mucosal barrier integrity, has been linked to systemic inflammatory consequences [18,19,26,27,28]. In the present case, the markedly elevated faecal alpha-1-antitrypsin may be compatible with mucosal barrier disturbance, whereas the faecal calprotectin value was only borderline elevated and should not be overinterpreted. Similarly, predominance of *E. coli* on conventional stool culture is a weak and non-specific finding, because routine culture cannot capture microbiome diversity, strain-level pathogenicity, virulence factors, or functional metabolic activity.

**Figure 3:**
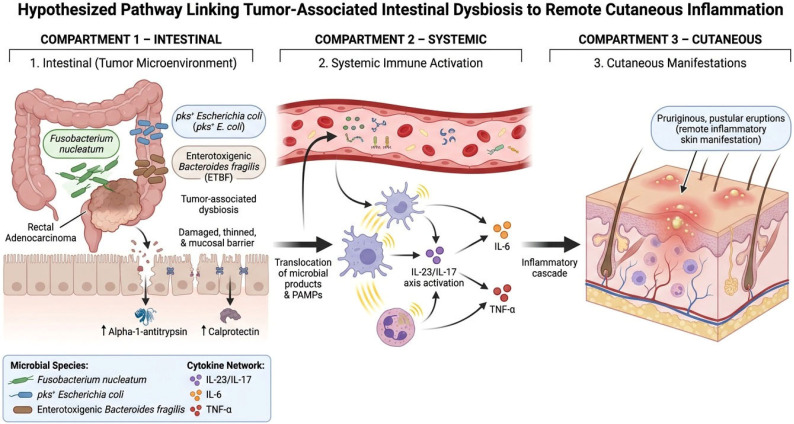
Hypothesised gut–skin–tumour axis linking rectal adenocarcinoma-associated intestinal alterations to remote cutaneous inflammation.

To our knowledge, the simultaneous documentation of these stool-based findings has not been previously reported in the context of a putative CRC-associated cutaneous eruption; however, this should be regarded as an observational detail rather than mechanistic evidence. Direct mechanistic validation in this patient—including circulating cytokines, tumour-associated mucosal microbiome, cutaneous microbiome, skin biopsy, or direct immunofluorescence—was not performed. The gut–skin–tumour axis is therefore offered strictly as an interpretive, hypothesis-generating framework, not as a demonstrated cascade. The hypothesised gut–skin–tumour pathway is illustrated in Figure 3.

Figure 3 is a schematic representation of a possible, hypothesis-generating pathway by which a rectal adenocarcinoma-associated intestinal microenvironment might contribute to systemic immune activation and cutaneous inflammatory manifestations. The intestinal compartment includes tumour-associated dysbiosis, with microbial taxa implicated in colorectal carcinogenesis in the literature, such as Fusobacterium nucleatum, pks+ Escherichia coli, and enterotoxigenic Bacteroides fragilis, together with mucosal barrier disturbance reflected by increased faecal alpha-1-antitrypsin and borderline faecal calprotectin. In the systemic compartment, translocation of microbial products and pathogen-associated molecular patterns may promote immune activation, including IL-23/IL-17 axis activation and inflammatory mediators such as IL-6 and TNF-α. In the cutaneous compartment, these systemic inflammatory signals are proposed to contribute to pruriginous/pustular inflammatory eruptions. These elements indicate concepts relevant to the present case and current literature; however, the pathway was not directly demonstrated in this patient by sequencing, tumour microbiome analysis, cytokine measurements, skin histopathology, or direct immunofluorescence. The systemic and cutaneous compartments are therefore conceptual rather than patient-demonstrated. The figure is schematic and hypothesis-generating. Concepts synthesised from references [14,15,18,29].

### 3.3. Faecal Haemoglobin as a Low-Cost Diagnostic Trigger

FIT for occult bleeding is a low-cost, non-invasive test endorsed by the 2023 NICE diagnostics guidance DG56 for symptomatic adults with suspected lower gastrointestinal pathology [20]. Multibiomarker stool studies have further supported the value of FIT-based triage in symptomatic populations [30]. In the present case, FIT positivity—identified during outpatient evaluation undertaken because of the unexplained dermatosis, not as part of a documented routine screening programme—redirected the diagnostic pathway toward colonoscopy and enabled the diagnosis of rectal cancer. This observation does not establish FIT as a general screening tool for paraneoplastic dermatoses, but raises a testable question: whether FIT could be evaluated prospectively as part of an individualised assessment of older patients with persistent, unexplained, treatment-resistant dermatoses, even in the absence of overt gastrointestinal alarm symptoms.

### 3.4. A Hypothesis-Generating Evaluation Framework

**Figure 4:**
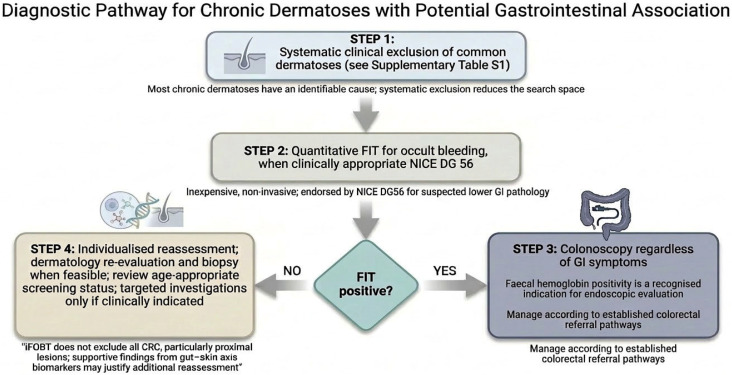
Hypothesis-generating diagnostic pathway for chronic treatment-resistant dermatoses with potential gastrointestinal association.

On the basis of this case and the contemporaneous literature, we propose a hypothesis-generating evaluation framework (Table 3, Figure 4) for older adults presenting with chronic, treatment-resistant dermatoses of unclear aetiology. The framework is intentionally conservative: it begins with individualised dermatologic reassessment and systematic exclusion of common mimickers, including biopsy or microbiological testing when clinically feasible, and reserves gastrointestinal investigation for patients with age-appropriate screening indications, positive FIT, or other concerning clinical features. Such a framework requires prospective validation before it can be regarded as a clinical recommendation; in its present form, it is intended to motivate study, not to direct practice.

The yield, cost-effectiveness, and stage-shift impact of such an approach in patients with treatment-refractory dermatoses of unclear aetiology are unknown and require prospective evaluation. An interrupted time-series or cluster-randomised design implemented across dermatology and internal medicine outpatient clinics would be a feasible study format.

Figure 4 represents the proposed evaluation framework for older adults presenting with chronic dermatoses of unclear aetiology after systematic clinical exclusion of common dermatologic causes. The pathway highlights quantitative faecal immunochemical testing (FIT) as a low-cost, non-invasive triage tool for occult lower gastrointestinal pathology in populations for which FIT is clinically appropriate and in line with established colorectal referral pathways. A positive FIT result should prompt colorectal evaluation according to established diagnostic pathways, irrespective of gastrointestinal symptom status. If FIT is negative, broad oncological imaging is not automatically indicated; instead, patients with persistent unexplained dermatoses should undergo individualised reassessment, dermatologic re-evaluation with biopsy when feasible, review of age-appropriate cancer screening status, and targeted investigations only if clinically indicated. The framework is hypothesis-generating and requires prospective validation before clinical implementation.

### 3.5. Strengths and Limitations

The principal strengths of this report are the well-documented oncological course, complete pathological response, prolonged disease-free follow-up, the parallel resolution of the cutaneous eruption without specific dermatologic intervention, and the integration of clinical, oncological, and stool-based findings within a cautious hypothesis-generating framework.

The principal limitation is the absence of a contemporaneous skin biopsy, which precludes histopathological characterisation of the dermatosis. No skin histopathology, direct immunofluorescence, dermoscopy, skin microbiological culture, circulating cytokine assessment, cutaneous microbiome analysis, or tumour-associated microbiome profiling was performed. Relevant dermatologic laboratory data, such as eosinophil count, IgE, and inflammatory markers, were not systematically available for analysis. To partly mitigate this gap, a comprehensive clinical differential diagnosis was systematically considered on the basis of morphological distribution, evolution, prior treatment failure, and the absence of supportive systemic or familial features (Supplementary Table S1). Common mimickers—including scabies, prurigo nodularis, bacterial folliculitis, dermatitis herpetiformis, cutaneous T-cell lymphoma, and small-vessel cutaneous vasculitis—were considered clinically less likely, but were not definitively excluded histologically. The descriptor “chronic pruriginous pustular eruption” therefore remains intentionally morphological. The paraneoplastic interpretation thus remains presumptive and hypothesis-generating, supported mainly by the temporal association with tumour-directed treatment and the absence of recurrence during prolonged follow-up. The gut–skin–tumour axis framework is offered as a hypothesis to be tested prospectively, not as a demonstrated mechanism in this single patient.

## 4. Conclusions

We report a case of locally advanced rectal adenocarcinoma in which an atypical, treatment-resistant pruriginous and pustular cutaneous eruption preceded the diagnosis in a patient without gastrointestinal alarm symptoms. Detection of faecal haemoglobin during outpatient clinical evaluation, undertaken because of the unexplained dermatologic findings, prompted colonoscopy and led to timely oncological diagnosis at a stage permitting curative-intent long-course neoadjuvant chemoradiotherapy, surgery with complete pathological response, and adjuvant capecitabine. The previously persistent cutaneous manifestations resolved during tumour-directed therapy, consistent with the temporal and parallel-course components of Curth’s criteria; however, the absence of skin biopsy and direct mechanistic data prevents causal attribution.

Pending formal validation, this case suggests that clinicians encountering patients aged ≥50 years with persistent, treatment-refractory dermatoses of unknown cause should ensure careful dermatologic reassessment, review age-appropriate cancer screening status, and consider FIT when colorectal evaluation is clinically appropriate.

A positive FIT result should be managed according to local colorectal referral pathways. Prospective studies are needed to determine whether incorporating FIT into the individualised assessment of unexplained dermatoses improves diagnostic timeliness, diagnostic yield, cost-effectiveness, or stage distribution.

Therefore, FIT should not be interpreted as a dermatological or paraneoplastic biomarker, but only as a colorectal cancer triage test that may be considered in selected patients when colorectal evaluation is otherwise clinically appropriate.

## Figures and Tables

**Table 1:** Stool-based biomarker findings during outpatient clinical assessment.

Parameter	Patient Result	Reference Range	Interpretation
Faecal haemoglobin (iFOBT)	Detected	Not detected	Decisive finding; indication for colonoscopy
Faecal calprotectin	51.6 mg/kg	≤50 mg/kg	Borderline elevation; non-specific finding
Faecal alpha-1-antitrypsin	631 µg/mL	≤270 µg/mL	Markedly elevated (~2.3× ULN); compatible with mucosal barrier disturbance
Pancreatic elastase	315 µg/g	≥200 µg/g	Preserved exocrine pancreatic function
Secretory IgA	Within range	510–2040 mg/L	Preserved mucosal humoral immunity
*E. coli* (variants)	1 × 10^7^ CFUs/g	≤1 × 10^6^ CFUs/g	Increased on conventional culture; non-specific and not a comprehensive microbiome profile
α-haemolytic streptococci	Increased	Low physiological	Possible mucosal inflammatory activity
Yeasts and moulds	Not detected	Not detected	No fungal overgrowth
Microbiome balance score	4	0–3	Mild dysbiosis

iFOBT, immunochemical faecal occult blood test; CFUs, colony-forming units; ULN, upper limit of normal.

**Table 2:** Selected cutaneous paraneoplastic syndromes associated with internal malignancy (illustrative, not exhaustive). Adapted from references [6,7,8,9,11,12].

Syndrome	Typical Morphology	Most Frequently Associated Malignancies
Acanthosis nigricans (malignant)	Symmetric hyperpigmented velvety plaques (axillae, neck)	Gastric, colorectal, and other GI adenocarcinomas
Tripe palms	Velvety thickening of palms with accentuated dermatoglyphics	Gastric, lung carcinoma
Sign of Leser–Trélat	Sudden eruption of multiple seborrhoeic keratoses	GI adenocarcinomas, lymphoproliferative disorders
Erythema gyratum repens	Migratory concentric serpiginous erythema	Lung, breast carcinoma
Necrolytic migratory erythema	Erosive annular erythema with crusting	Glucagonoma
Paraneoplastic pemphigus	Polymorphous mucocutaneous blistering and erosions	Lymphoma, Castleman disease, thymoma
Paraneoplastic dermatomyositis	Heliotrope rash, Gottron papules, proximal myopathy	Ovarian, lung, gastric, colorectal
Sweet syndrome (paraneoplastic)	Acute febrile tender erythematous plaques with neutrophilic infiltrate	Acute myeloid leukaemia, solid tumours
Pyoderma gangrenosum (paraneoplastic)	Painful ulcer with violaceous undermined border	Haematological malignancies, GI cancers
Atypical non-classical pruriginous/pustular eruptions are reported in isolated cases	Heterogeneous; refractory pruritus, pustules, crusting	Rare and heterogeneous; hypothesis-generating observations only

**Table 3:** Hypothesis-generating evaluation framework for older adults presenting with chronic, treatment-resistant dermatoses of unclear aetiology. The framework requires prospective validation before being regarded as a clinical recommendation.

Step	Action	Rationale
1	Systematic clinical exclusion of common dermatoses (see Supplementary Table S1)	Most chronic dermatoses have an identifiable cause; systematic exclusion reduces the search space
2	Quantitative faecal immunochemical test (FIT) for occult bleeding	Inexpensive, non-invasive, endorsed by NICE DG56 for suspected lower GI pathology
3	If FIT positive → managed according to established colorectal referral pathways	FIT positivity generally justifies colorectal evaluation in appropriate clinical contexts
4	Negative FIT: individualised clinical reassessment; dermatology re-evaluation and skin biopsy when feasible; targeted investigations only if clinically indicated	Negative FIT: does not fully exclude CRC, particularly proximal lesions; consider individualised reassessment based on clinical context, screening status, and persistent concerning features

GI, gastrointestinal; CRC, colorectal cancer; FIT, faecal immunochemical test.

## Data Availability

The data supporting the findings of this study are included within the article. Additional anonymized information may be made available by the corresponding author upon reasonable request, subject to ethical and privacy restrictions related to patient confidentiality.

## Supplementary Materials

The following supporting information can be downloaded at: https://www.mdpi.com/article/10.3390/diagnostics16111592/s1, Table S1: systematic clinical differential diagnosis considered for the chronic pruriginous, pustular, and crusted eruption.

## References

[1] Bray F., Laversanne M., Sung H., Ferlay J., Siegel R.L., Soerjomataram I., Jemal A. (2024). Global cancer statistics 2022: GLOBOCAN estimates of incidence and mortality worldwide for 36 cancers in 185 countries. CA Cancer J. Clin..

[2] Siegel R.L., Giaquinto A.N., Jemal A. (2024). Cancer statistics, 2024. CA Cancer J. Clin..

[3] Dekker E., Tanis P.J., Vleugels J.L.A., Kasi P.M., Wallace M.B. (2019). Colorectal cancer. Lancet.

[4] Suryani N.D., Wiranata J.A., Puspitaningtyas H., Hutajulu S.H., Prabandari Y.S., Handaya A.Y., Hardianti M.S., Taroeno-Hariadi K.W., Kurnianda J., Purwanto I. (2024). Determining factors of presentation and diagnosis delays in patients with colorectal cancer and the impact on stage: A cross-sectional study in Yogyakarta, Indonesia. Ecancermedicalscience.

[5] Weller D., Vedsted P., Rubin G., Walter F.M., Emery J., Scott S., Campbell C., Andersen R.S., Hamilton W., Olesen F. (2012). The Aarhus statement: Improving design and reporting of studies on early cancer diagnosis. Br. J. Cancer.

[6] Pelosof L.C., Gerber D.E. (2010). Paraneoplastic syndromes: An approach to diagnosis and treatment. Mayo Clin. Proc..

[7] Thiers B.H., Sahn R.E., Callen J.P. (2009). Cutaneous manifestations of internal malignancy. CA Cancer J. Clin..

[8] Silva J.A.D., Mesquita K.D.C., Igreja A.C.D.S.M., Lucas I.C.R.N., Freitas A.F., Oliveira S.M.D., Costa I.M.C., Campbell I.T. (2013). Paraneoplastic cutaneous manifestations: Concepts and updates. An. Bras. Dermatol..

[9] Wong C.Y., Helm M.A., Kalb R.E., Helm T.N., Zeitouni N.C. (2013). The presentation, pathology, and current management strategies of cutaneous metastasis. N. Am. J. Med. Sci..

[10] Curth H.O., Andrade R., Gumport S.L., Popkin G.L., Rees T.D. (1976). Skin lesions and internal carcinoma. Cancer of the Skin: Biology, Diagnosis, Management.

[11] Ehst B.D., Minzer-Conzetti K., Swerdlin A., Devere T.S. (2010). Cutaneous manifestations of internal malignancy. Curr. Probl. Surg..

[12] Didona D., Fania L., Didona B., Eming R., Hertl M., Di Zenzo G. (2020). Paraneoplastic dermatoses: A brief general review and an extensive analysis of paraneoplastic pemphigus and paraneoplastic dermatomyositis. Int. J. Mol. Sci..

[13] Yuste-Chaves M., Unamuno-Pérez P. (2013). Cutaneous alerts in systemic malignancy: Part I. Actas Dermosifiliogr..

[14] Diaconu C., Gheorghe G., Bacalbaşa N., Stan-Ilie M., Ionescu V.A. (2025). Cutaneous paraneoplastic syndromes in colorectal cancer patients. Gastrointest. Disord..

[15] De Pessemier B., Grine L., Debaere M., Maes A., Paetzold B., Callewaert C. (2021). Gut–skin axis: Current knowledge of the interrelationship between microbial dysbiosis and skin conditions. Microorganisms.

[16] Sinha S., Lin G., Ferenczi K. (2021). The skin microbiome and the gut–skin axis. Clin. Dermatol..

[17] Mahmud R., Akter S., Tamanna S.K., Mazumder L., Esti I.Z., Banerjee S., Akter S., Hasan R., Acharjee M., Hossain S. (2022). Impact of gut microbiome on skin health: Gut–skin axis observed through the lenses of therapeutics and skin diseases. Gut Microbes.

[18] Brennan C.A., Garrett W.S. (2019). Fusobacterium nucleatum—Symbiont, opportunist and oncobacterium. Nat. Rev. Microbiol..

[19] Yang Y., Du L., Shi D., Kong C., Liu J., Liu G., Li X., Ma Y. (2021). Dysbiosis of human gut microbiome in young-onset colorectal cancer patients. Nat. Commun..

[20] National Institute for Health and Care Excellence (2023). Quantitative faecal immunochemical testing to guide colorectal cancer pathway referral in primary care. Diagnostics Guidance DG56.

[21] Bahadoer R.R., Dijkstra E.A., van Etten B., Marijnen C.A.M., Putter H., Kranenbarg E.M.-K., Roodvoets A.G.H., Nagtegaal I.D., Beets-Tan R.G.H., Blomqvist L.K. (2021). Short-course radiotherapy followed by chemotherapy before total mesorectal excision (TME) versus preoperative chemoradiotherapy, TME, and optional adjuvant chemotherapy in locally advanced rectal cancer (RAPIDO): A randomised, open-label, phase 3 trial. Lancet Oncol..

[22] Conroy T., Bosset J.-F., Etienne P.-L., Rio E., François E., Mesgouez-Nebout N., Vendrely V., Artignan X., Bouché O., Gargot D. (2021). Neoadjuvant chemotherapy with FOLFIRINOX and preoperative chemoradiotherapy for patients with locally advanced rectal cancer (UNICANCER-PRODIGE 23): A multicentre, randomised, open-label, phase 3 trial. Lancet Oncol..

[23] Mechera R., Schuster T., Rosenberg R., Speich B. (2017). Lymph node yield after rectal resection in patients treated with neoadjuvant radiation for rectal cancer: A systematic review and meta-analysis. Eur. J. Cancer.

[24] Marschner M.L., Reinhardt J.F. (2011). Malignant acanthosis nigricans in rectal adenocarcinoma. Del. Med. J..

[25] Bittencourt M.J.S., Imbiriba A.A., Oliveira O.A., Santos J.E.B.D. (2018). Cutaneous metastasis of colorectal cancer. An. Bras. Dermatol..

[26] Kostic A.D., Chun E., Robertson L., Glickman J.N., Gallini C.A., Michaud M., Clancy T.E., Chung D.C., Lochhead P., Hold G.L. (2013). Fusobacterium nucleatum potentiates intestinal tumorigenesis and modulates the tumor-immune microenvironment. Cell Host Microbe.

[27] Shigematsu Y., Saito R., Amori G., Kanda H., Takahashi Y., Takeuchi K., Takahashi S., Inamura K. (2024). Fusobacterium nucleatum, immune responses, and metastatic organ diversity in colorectal cancer liver metastasis. Cancer Sci..

[28] Schinocca C., Rizzo C., Fasano S., Grasso G., La Barbera L., Ciccia F., Guggino G. (2021). Role of the IL-23/IL-17 pathway in rheumatic diseases: An overview. Front. Immunol..

[29] Liu J., Duan Y., Cheng X., Chen X., Xie W., Long H., Lin Z., Zhu B. (2011). IL-17 is associated with poor prognosis and promotes angiogenesis via stimulating VEGF production of cancer cells in colorectal carcinoma. Biochem. Biophys. Res. Commun..

[30] Hijos-Mallada G., Saura N., Lué A., Velamazan R., Nieto R., Navarro M., Arechavaleta S., Chueca E., Gomollon F., Lanas A. (2023). A Point-of-Care Faecal Test Combining Four Biomarkers Allows Avoidance of Normal Colonoscopies and Prioritizes Symptomatic Patients with a High Risk of Colorectal Cancer. Cancers.

