# Supplementary material for: Atypical Pruriginous Pustular Eruption Preceding Locally Advanced Rectal Cancer: A Case Report and Gut–Skin–Tumour Axis Hypothesis
Source: Diagnostics (Basel). 2026 May 22;16(11):1592. doi: 10.3390/diagnostics16111592 (PMC13256220; doi:10.3390/diagnostics16111592)
Supplement: Supplementary file 1 [file diagnostics-16-01592-s001.zip › diagnostics-4324098-supplementary.pdf]

diagnostics MDPI

Supplementary Table S1. Systematic clinical differential diagnosis was considered for the chronic pruriginous, pustular, and crusted symmetric cutaneous eruption documented at outpatient evaluation.

Considered Entity	Key Clinical Features Expected	Reasoning for Exclusion in this Patient
Scabies	Severe nocturnal pruritus, interdigital and flexural burrows, household contacts affected	No nocturnal pruritus accentuation; no household members with similar lesions; distribution dorsal symmetric rather than flexural; empirical scabicidal treatment was not documented
Prurigo nodularis	Discrete, hard, hyperpigmented excoriated nodules secondary to chronic scratching	Predominantly pustular and crusted morphology rather than firm excoriated nodules; the symmetric distribution was less typical of classic prurigo nodularis.
Bacterial folliculitis	Follicular pustules; positive bacterial cultures; response to antibacterial therapy	Symmetric distribution on extensor surfaces of all four limbs uncommon for folliculitis; partial course of oral antibiotics did not produce sustained response
Dermatitis herpetiformis	Intensely pruritic vesicles and papulovesicles on extensor surfaces; gluten-sensitive enteropathy; granular IgA deposits at the dermoepidermal junction	Predominantly pustular and crusted morphology rather than tense vesicles; absence of clinical features suggesting coeliac enteropathy; not a classical CRC association
Cutaneous T-cell lymphoma (mycosis fungoides, early)	Chronic patches/plaques, often photoprotected sites; gradual progression over years; histology with epidermotropism	Limited limb-only distribution and short 5-month evolution atypical; complete resolution under antineoplastic therapy aimed at rectal cancer not consistent with cutaneous T-cell lymphoma trajectory
Small-vessel cutaneous vasculitis	Palpable purpura, often dependent areas; possible systemic involvement; histology with leukocytoclastic vasculitis	Absence of palpable purpura; absence of fever or systemic inflammatory features; symmetric distribution but pustular rather than purpuric morphology
Allergic contact dermatitis / drug eruption	Temporal association with new exposure; eczematous or urticated morphology; resolution after withdrawal	No identified novel topical exposure, sensitiser, or systemic medication temporally related to onset; pustular rather than eczematous morphology

IgA, immunoglobulin A. The systematic exclusion of common alternative diagnoses partly compensates for the absence of contemporaneous skin biopsy; the descriptor “chronic pruriginous pustular eruption” is intentionally morphological.
